# The "Nail File Sign": A Report of Three Cases With Varied Etiologies

**DOI:** 10.7759/cureus.95996

**Published:** 2025-11-03

**Authors:** Vasudeva G Iyer, Lisa B Shields, Yi Ping Zhang, Christopher B Shields

**Affiliations:** 1 Clinical Neurophysiology, Neurodiagnostic Center of Louisville, Louisville, USA; 2 Norton Neuroscience Institute, Norton Healthcare, Louisville, USA

**Keywords:** cubital tunnel syndrome, electrodiagnostic studies, nail file sign, neurology, ulnar nerve, ulnar neuropathy, ultrasound studies

## Abstract

Similar to the Wartenberg, Froment, and Duchenne signs, the more recently described "nail file sign" is also considered a diagnostic feature of ulnar nerve neuropathy. The "nail file sign" indicates a patient's inability to maintain the distal interphalangeal (DIP) joints of the small and ring fingers in a flexed position for filing the nail. Localizing the site of an ulnar nerve lesion is based on the topography of muscle weakness. The presence of the "nail file sign" points to a location at or proximal to the branch of the flexor digitorum profundus (FDP) muscle. We describe three patients who exhibited the "nail file sign," each with a different etiology. One patient had an ulnar nerve neuropathy at the elbow from compression by a cyst, another had ulnar neuropathy from entrapment at the cubital tunnel, and the third had it from an injury to the FDP tendons attached to the small and ring fingers. Testing for weakness of flexion at the DIP joints of the ring and small fingers should be an essential part of the clinical evaluation of patients with ulnar neuropathy, as it provides an important clue for anatomic localization of the lesion. Ultrasound studies complement the electrodiagnostic evaluation, not only to confirm the location but also to determine the cause of the ulnar nerve neuropathy.

## Introduction

The ulnar nerve arises from the medial cord of the brachial plexus and the C8 and T1 nerve roots [1-3]. It provides motor innervation to the flexor carpi ulnaris (FCU), the ulnar half of the flexor digitorum profundus (FDP) muscles in the forearm, and many intrinsic hand muscles. It also provides sensory cutaneous innervation to the medial aspect of the wrist, medial palm and dorsum of the hand, and the small finger and ulnar side of the ring finger. The ulnar nerve is prone to entrapment (most often at the cubital tunnel at the elbow and Guyon's canal at the wrist) or extrinsic compression (where the nerve is superficial) [1]. The ulnar nerve may also sustain trauma (fractures, dislocations, or lacerations) or repetitive stress injuries [4]. Ulnar nerve neuropathy at the elbow is the second most common focal neuropathy of the upper extremity, following median nerve entrapment at the carpal tunnel.

The classic signs of ulnar nerve neuropathy, such as the Wartenberg sign (weak adduction leading to an abducted posture of the small finger caused by unopposed action of the extensor digiti minimi muscle), the Froment sign (hyperflexion at the interphalangeal (IP) joint of the thumb on holding a paper between the thumb and index finger due to the flexor pollicis longus (FPL) muscle compensating for a weak adductor pollicis (AdP) muscle), and the Duchenne sign or claw deformity (ring and little fingers are hyperextended at the metacarpophalangeal joints and flexed at the IP joints) may occur with lesions at distal and proximal sites of the ulnar nerve involvement [3,5,6]. The Duchenne sign usually results from lesions at distal sites associated with ulnar nerve involvement. However, some conditions affect a few fascicles more than others; hence, a proximal lesion may result in signs typical of a distal lesion, a well-known pitfall in clinical diagnosis. Documentation of weakness in the FDP muscle suggests a more proximal site of involvement, most often at the elbow.

In 1919, Pollock described weakness of the FDP muscle in ulnar neuropathy [7]. He reported that "flexion of the distal and proximal phalanges of the ring and little fingers is performed by the two inner tendons of flexor profundus digitorum and the two lumbricales, respectively. The imperfect flexion of these phalanges is the result of the influence exerted on all segments when the flexor sublimus digitorum contracts. This is more marked in the little finger than in the ring finger" [7]. Selective weakness of flexion of the distal interphalangeal (DIP) joint of the small and ring fingers (Pollock's sign) localizes ulnar neuropathy at or proximal to the innervation of the FDP muscle [7]. The radial half of the FDP muscle is innervated by the anterior interosseous branch of the median nerve, and the ulnar half by the ulnar nerve. Flexion of the DIP joints of the index and middle fingers is intact, while that of the ring and small fingers is weak in proximal ulnar neuropathies. A sports-related injury to the FDP tendon resulting in jersey finger (most often affecting the ring finger) as well as a spontaneous rupture of the FDP tendon, which is idiopathic or related to conditions such as rheumatoid arthritis or deformity of the DIP in an extended position due to arthritis, can result in a clinical presentation similar to the "nail file sign" reported in ulnar nerve palsy.

The more recent nomenclature "failing ulnar hook," "ulnar clasp failure," and the "nail file sign" refer to an inability to file the nail of the small finger due to the inability to flex the DIP joint, resulting from weakness in the ulnar portion of the FDP muscle [8]. Herein, we report three cases of the "nail file sign" with diverse etiologies. The physical examination, as well as electrodiagnostic (EDX) and ultrasound (US) studies to localize the lesions and determine the causes, are discussed.

## Case presentation

Case 1

History and Physical Examination

**Figure 1:**
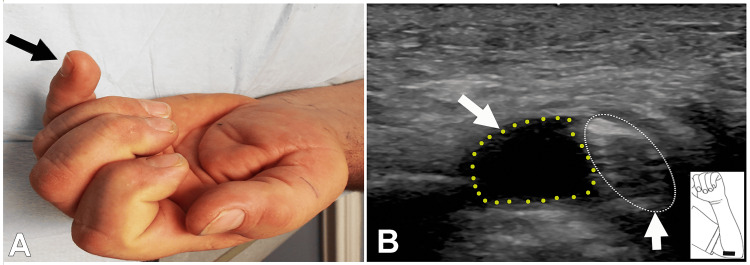
"Nail File Sign" and a Cyst at the Elbow Compressing the Ulnar Nerve Detected on an Ultrasound Study Case 1: (A) "Nail file sign" of the left hand: inability to flex the small finger at the distal interphalangeal joint (arrow). (B) Ultrasound study demonstrating a cyst at the elbow compressing the ulnar nerve (vertical arrows point to the ulnar nerve; oblique arrow points to the cyst).

A 54-year-old male presented with weakness of the left hand, pain at the elbow, and numbness of the small finger. He sustained an injury to the left upper extremity in childhood and experienced limitations of extension at the elbow as well as intermittent pain and paresthesia in the left hand. Following a recent blunt injury to the posterior aspect of the left elbow, he noted persistent numbness in the small finger and weakness in the left hand. Wasting of the left first dorsal interosseous (FDI) and abductor digiti minimi (ADM) muscles was detected on exam. There was also marked weakness of the FDP muscle, causing poor flexion at the DIP joint of the small finger, which exhibited the "nail file sign" (Figure 1A), and to a lesser extent, of the ring finger. Additionally, the FCU, FDI, and ADM muscles were weak. Pinprick and light touch sensations were impaired in the ulnar nerve distribution. Both flexion and extension of the left elbow were limited with tenderness over the ulnar nerve.

EDX and US Studies

A nerve conduction study demonstrated marked slowing of motor conduction at the elbow with loss of sensory potentials over the digital and dorsal cutaneous branches of the ulnar nerve. Fibrillations were noted in the FDI, ADM, and FDP (ulnar) muscles, with large-amplitude, wide-duration motor unit potentials (MUPs), and decreased MUP recruitment. A US study revealed an enlarged ulnar nerve at the elbow, with a cyst compressing the nerve (Figure 1B). The ganglion cyst was excised along with transposition of the ulnar nerve, nerve transfer involving a branch of the anterior interosseous nerve (AIN) to the ulnar nerve, and side-to-side transfer of the FDP tendon of the middle finger to the ring and small fingers.

Case 2

History and Physical Examination

**Figure 2:**
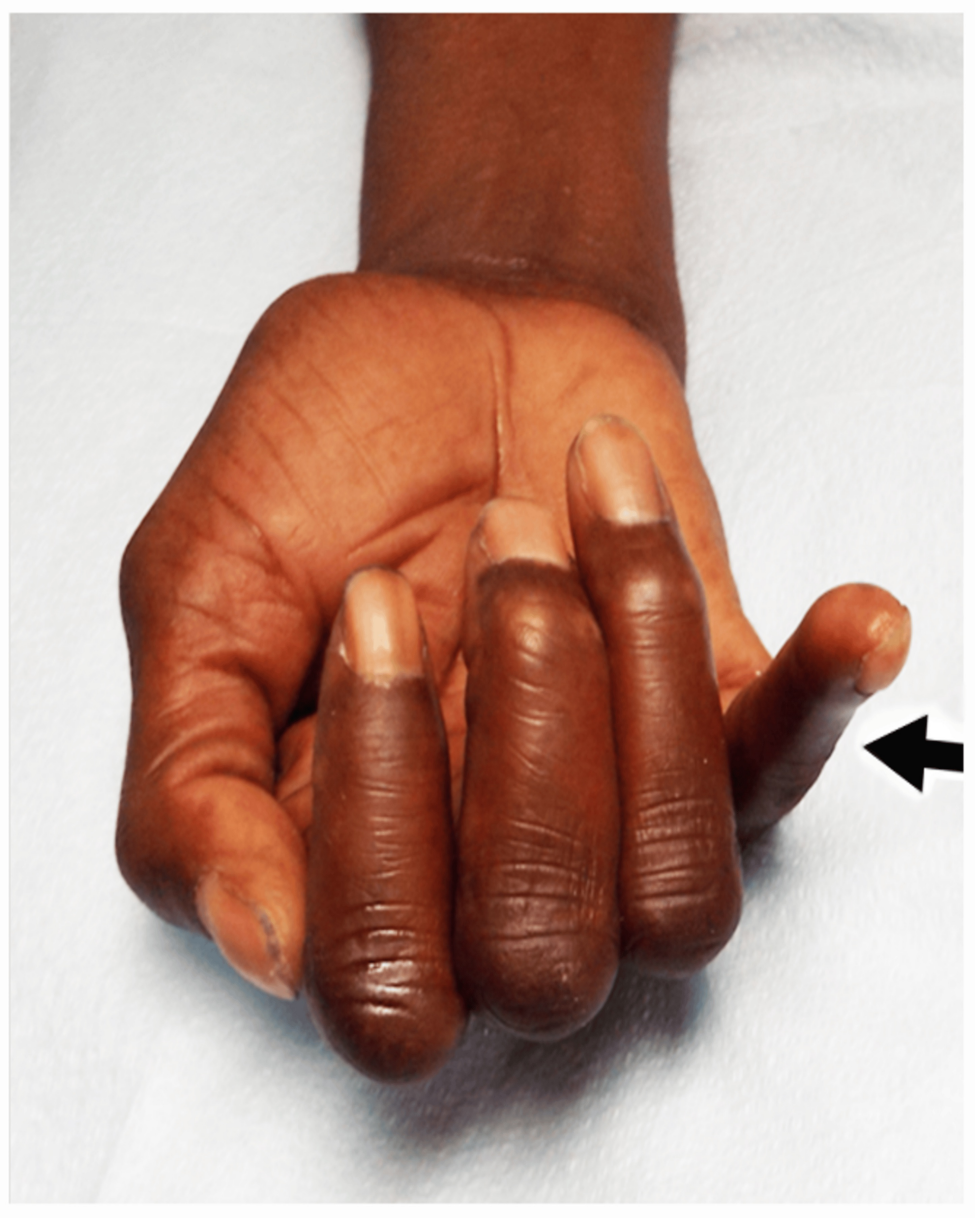
"Nail File Sign" Case 2: The "nail file sign" of the right hand. The arrow points to the small finger with a lack of flexion in the distal interphalangeal (DIP) joint.

A 54-year-old female presented with a history of weakness and atrophy of the muscles of the right hand and numbness of the small and ring fingers. She denied trauma to the right elbow or wrist. The "nail file sign" was noted on the right hand (Figure 2). There was a significant weakness in the right ADM, FDI, and FDP (small finger) muscles. Pinprick sensation was decreased in the small finger, as well as on the ulnar side of both the palm and the dorsum of the hand.

EDX and US Studies

**Figure 3:**
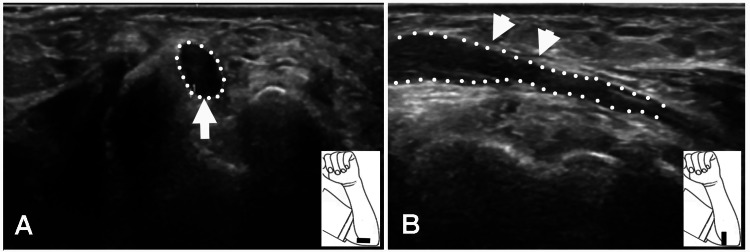
Ulnar Nerve on Ultrasound Studies Case 2: (A) Large hypoechoic ulnar nerve at the elbow (arrow). The arrow indicates the large diameter of the ulnar nerve. (B) A decrease in the diameter of the ulnar nerve in the cubital tunnel. The two arrows show the decreased diameter of the ulnar nerve.

A motor conduction study demonstrated marked slowing of motor conduction of the right ulnar nerve across the elbow and, to a lesser extent, in the forearm. No sensory nerve action potentials (SNAP) were noted over the digital or dorsal cutaneous branches of the ulnar nerve. Needle EMG showed one to two large-amplitude, wide-duration MUPs in the ADM and FDI muscles. The likely location of the lesion was the elbow, with focal and anterograde demyelination to explain the slowing of motor conduction in the forearm, distal to the cubital tunnel. A US study revealed a large hypoechoic ulnar nerve at the elbow with a decrease in diameter within the cubital tunnel (Figures 3A, 3B).

Case 3

History and Physical Examination

**Figure 4:**
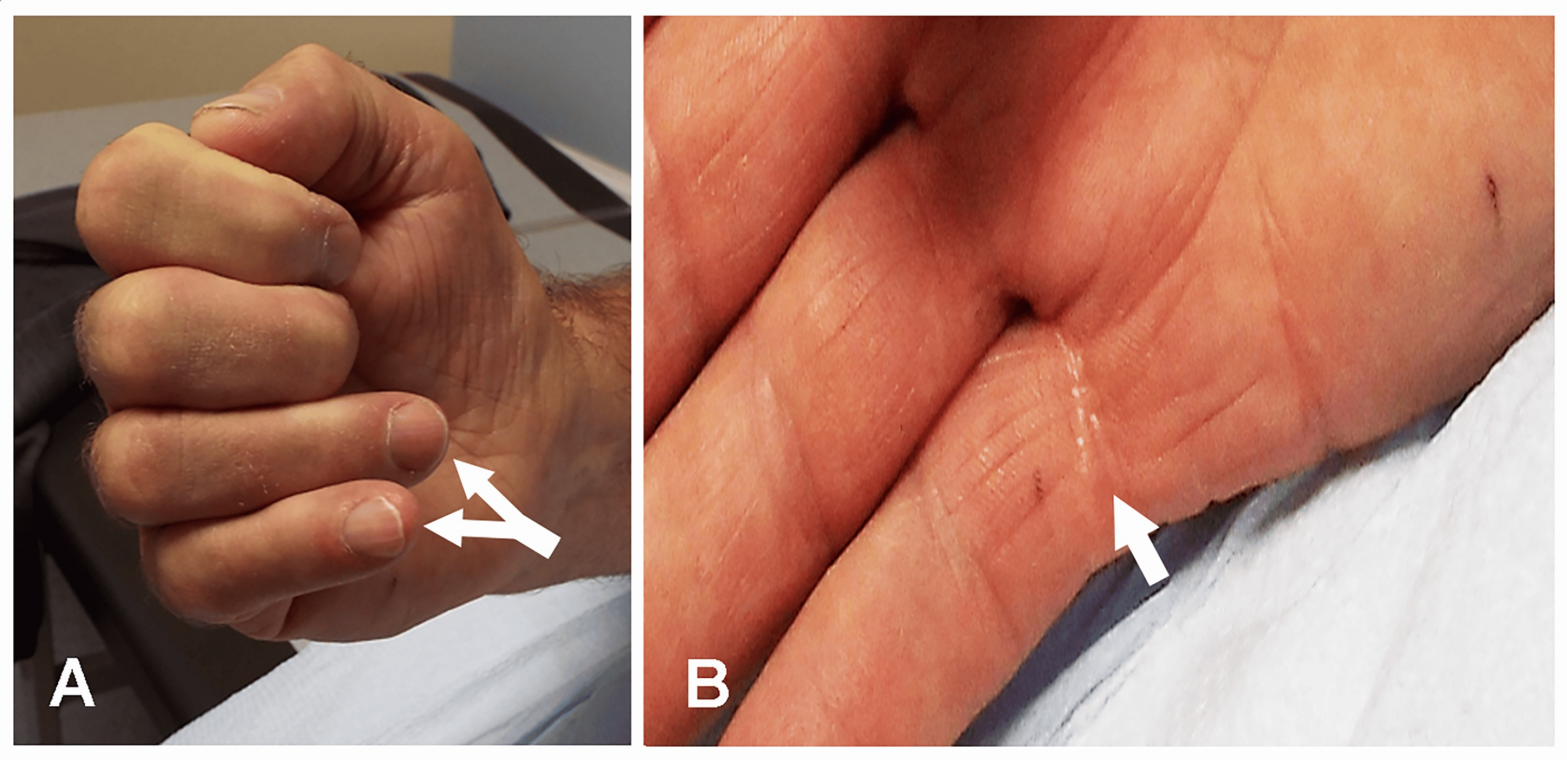
"Nail File Sign" and Scar From a Laceration Injury Case 3: (A) The "nail file sign" of the right hand with an inability to flex the DIP joint of the small finger and partial weakness of flexion of the distal interphalangeal (DIP) joint of the ring finger. (B) Scar from an old laceration injury to the flexor digitorum profundus tendon at the base of the ring and small fingers, resulting in an inability to flex the interphalangeal joints of these fingers.

A 60-year-old diabetic male with a history of numbness and coldness in his right hand was referred for EDX studies. Past history was significant for a laceration injury at the base of the ring and the small fingers, with an inability to flex the IP joints of these fingers. There was weakness of flexion of the DIP joints of the small and ring fingers (Figures 4A, 4B). Atrophy of the right thenar muscles, with severe weakness of the APB muscle, was noted. The patient was unable to flex the DIP joints of the ring and small fingers. The FDI and ADM muscles were normal.

EDX and US Studies

Nerve conduction studies demonstrated normal motor conduction of the right ulnar nerve with the recording electrode placed over the ADM and FDI muscles. The SNAPs were normal. Stimulation of the right median nerve did not evoke compound muscle action potentials over the APB muscle. With the recording electrode placed over the second lumbrical muscle, the distal motor latency was prolonged with a normal motor conduction velocity across the forearm. Needle EMG revealed normal recruitment of MUPs in the ADM, FDI, and FDP muscles. The APB muscle showed denervation changes with no motor units. A US study detected a significant increase in the cross-sectional area (CSA) of the median nerve at the carpal tunnel inlet and a normal CSA in the mid-forearm. These findings suggested an entrapment at the carpal tunnel. The ulnar nerve was normal at the wrist and elbow. The "nail file sign" was due to injury to the FDP tendons at the base of the small and ring fingers and not from ulnar neuropathy.

Table 1 summarizes the nerve conduction and EMG findings, as well as the US features, for the three cases in our series.

**Table 1:** Nerve Conduction, EMG Findings, and US Features of the Three Cases CSA at the level of the medial epicondyle: Normal < 10 mm² DL: distal latency; CV: conduction velocity; CMAP: compound motor action potentials; SNAP: sensory nerve action potentials; Fibs: fibrillations; PSW: positive sharp waves; MUP: motor unit potentials; CSA: cross-sectional area; NR: not recorded; Y: yes; N: no Table Credits: Vasudeva G. Iyer, M.D.

Case #	DL (ms)	CV in the Forearm	CV Across the Elbow	CMAP Amplitude (mV)	SNAP Lat (ms)/amp (µV)	Fibs/PSW	Increase in Polyphasic MUP	Decrease in MUP Recruitment	CSA at the Level of Medial Epicondyle (mm^2^)
1	3.5	31.5	31.1	4.4	NR	1+	Y	Y	23
2	4.5	32.0	20.0	1.41	NR	No	Y	Y	26
3	3.0	58.7	67.4	9.8	3.1/29.3	No	No	No	9

## Discussion

Coining the terms "failing ulnar hook," "ulnar clasp failure," and "nail file sign" for failure of flexion at the DIP joints of the small and ring fingers, Kapandji described a female patient who was unable to file the nail of her small finger due to weakness of the FDP muscle [8]. That author also described a technique to evaluate the FDP muscle by asking the patient to roll the small and ring fingers around the examiner's index finger and resist extending these fingers using the index finger of the examiner's other hand [8]. The "nail file sign" and Pollock's sign are important for confirming and localizing ulnar nerve palsy. When the sign is present, the lesion is located at or proximal to the branch from the ulnar nerve to the FDP muscle. When the sign is absent and other signs of ulnar nerve palsy are apparent, it suggests a more distal palsy. Goloborod'ko reported a different technique for testing the FDP muscle: the patient was instructed to actively flex the small fingers bilaterally and then pull the joined small fingers against each other [9]. Weakness of the FDP muscle on the affected hand was confirmed if the distal and middle phalanges gave way on the affected hand.

**Figure 5:**
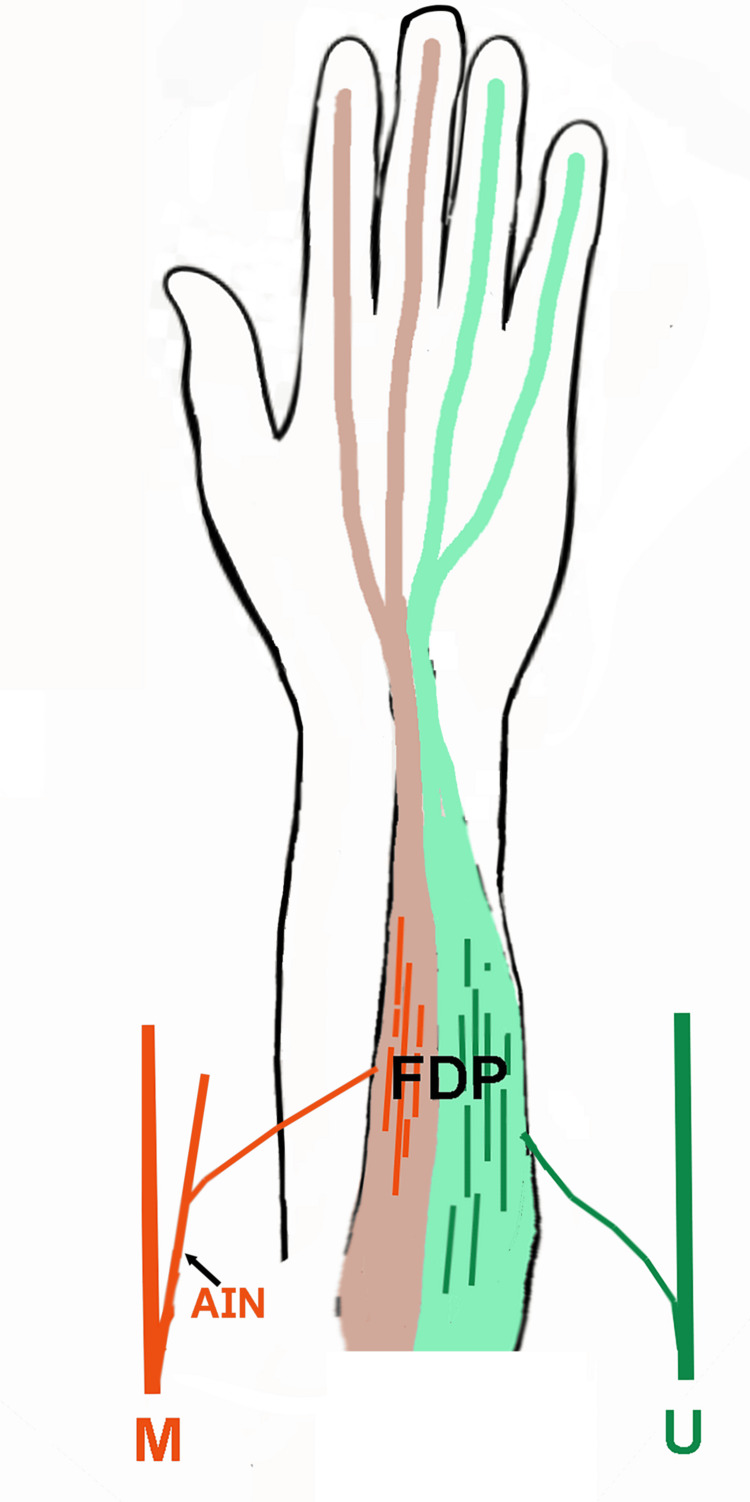
Dual Innervation of the Flexor Digitorum Profundus Muscle Drawing of the volar view of the hand depicting the dual innervation of the flexor digitorum profundus (FDP) muscle: the ulnar (U) nerve innervates the ulnar half of the muscle flexing the interphalangeal (IP) joint of the ring and small fingers (green), and the anterior interosseous branch of the median (M) nerve innervates the radial half of the muscle flexing the IP joint of the index and middle fingers (pink). Image Credits: Yi Ping Zhang, M.D. Source from which the information in this image was derived [10].

The medial half of the FDP muscle is innervated by the ulnar nerve and the lateral half by the anterior interosseous branch of the median nerve (Figure 5) [10]. This dual innervation results in weakness of flexion of the DIP joints of the ring and small fingers following the ulnar nerve lesions proximal to the origin of the branch to the FDP muscle. In proximal median nerve/AIN lesions, flexion of the DIP joints of the index and middle fingers will be selectively weak. However, variations in the innervation pattern to the FDP muscle can make precise localization difficult. In their review of the innervation of the FDP muscle, Hwang and colleagues reported that the FDP muscle of the small finger was innervated by the ulnar nerve alone in 64.6% of cases, while 35.4% received dual innervation from the AIN and ulnar nerve [10]. The ring FDP muscle was innervated by the ulnar nerve only in 85.4% of cases and by the ulnar nerve and AIN in 14.6% of cases. The FDP muscle of the middle finger received dual innervation from both the ulnar and AIN nerves in 76.8% of cases.

The Pollock and "nail file" signs indicate selective weakness of the FDP muscles of the small and ring fingers, suggesting proximal ulnar neuropathy. Table 2 summarizes different techniques used to clinically document this specific muscle weakness. While the findings are of significant localizing value, not every case of proximal ulnar neuropathy demonstrates the "nail file sign." One possibility is that patients with entrapment neuropathies may not have all nerve fascicles of the ulnar nerve uniformly affected. When the fascicles that supply the FDP muscle are spared due to either their topography at the site of entrapment or the extent of ischemia from neurovascular involvement, weakness of the FDP muscles of the ring and small fingers may not occur. Case 3 in our series illustrates that the "nail file sign" is not necessarily pathognomonic of proximal ulnar nerve neuropathy. Similar clinical findings may be observed following an injury to the FDP tendons. A distinction can be made by documenting normal EDX findings, as in our Case 3. One should be cognizant of the rare occurrence of a "pseudo nail file sign" (not caused by ulnar nerve neuropathy) similar to reported cases of "pseudo Wartenberg sign" [5]. Careful clinical examination can distinguish between true "nail file sign" secondary to ulnar neuropathy and "pseudo nail file sign" secondary to conditions like FDP tendon rupture. One should look for weakness in ulnar nerve-innervated muscles and sensory loss in the ulnar nerve distribution. Cases 1 and 2 clearly showed features of ulnar neuropathy, unlike Case 3. Nerve conduction and EMG studies are necessary to confirm ulnar neuropathy, localize it, and obtain insight into the underlying process, such as demyelination, conduction block, and axon loss.

**Table 2:** Clinically Testing the Strength of the Flexor Digitorum Profundus Muscle DIP: distal interphalangeal Table Credits: Vasudeva G. Iyer, M.D.

Clinically Testing the Strength of the Flexor Digitorum Profundus Muscle	References
(1) Fully contract the muscle (flex at the DIP joints) and maintain the contraction against force (resist extension of the DIP joints by the examiner)	[8,11,12]
(2) Contract the muscle against force (try to flex the DIP joint against the opposing force applied by the examiner)	[8,11,12]
(3) Compare with the asymptomatic side (patient makes a hook by flexing the DIP joints on both sides and pulls against each other)	[8,11,12]

A thorough clinical evaluation is the first step in localizing the site of an ulnar nerve lesion. In addition to sensory testing, testing the strength of the FDP, FCU, ADM, AdP, and FDI muscles should be performed. The presence of clinical signs such as Wartenberg's, Froment's, and Duchenne's should be noted. In cases of distal ulnar neuropathy presenting with selective weakness of the ADM and FDI muscles, the palmaris brevis sign (intact contraction of the palmaris brevis muscle causing puckering of the hypothenar skin) localizes the lesion to the deep branch of the ulnar nerve [13]. EDX studies of the ulnar nerve should then be conducted, which should include comprehensive motor tests by placing recording electrodes over the ADM and FDI muscles (including short segment studies across the elbow), sensory tests (both digital and dorsal cutaneous branches), and needle EMG of the FDI, ADM, FDP, and FCU muscles.

Our cases also illustrate the value of including the US in the evaluation of ulnar neuropathy. In Case 1, the US detected a ganglion cyst in the proximity of the ulnar nerve, which provided the correct diagnosis and assisted in formulating the optimal surgical procedure. In Case 2, the US study supported the diagnosis of cubital tunnel syndrome.

## Conclusions

The "nail file sign" resulting from a patient's inability to maintain the DIP joint in a flexed position usually indicates an ulnar nerve lesion at or proximal to the innervation of the FDP muscle. We present three patients with the "nail file sign," each with a different etiology. One patient had an ulnar nerve neuropathy at the elbow from compression by a ganglion cyst, another had ulnar nerve entrapment at the cubital tunnel, and the third had it from injury to the medial tendons of the FDP muscle distally. Detecting weakness of flexion at the DIP joints of the ring and small fingers is helpful for anatomic localization of an ulnar nerve lesion and should be part of the routine clinical evaluation. EDX and US evaluations are valuable in localizing and determining the underlying etiology of an ulnar nerve neuropathy.
